# ADDing up?

**DOI:** 10.1177/10398562231211125

**Published:** 2023-10-30

**Authors:** Gordon Parker

**Affiliations:** Discipline of Psychiatry & Mental Health, School of Clinical Medicine, 7800 University of New South Wales, Sydney, NSW, Australia

**Keywords:** attention-deficit disorder, attention-deficit hyperactivity disorder, late-onset attention-deficit hyperactivity disorder

## Abstract

**Objective:**

To hypothesise that a possible increased prevalence of adult-onset attention-deficit disorder (ADD) and attention-deficit hyperactivity disorder (ADHD) may reflect mobile technology and digital media use being an aetiological factor.

**Conclusions:**

Data and clinical observations support but do not prove the hypothesis.

It is common for clinical psychiatrists to comment that they are noting a distinct increase in adolescent and adult patients presenting with ADD (attention-deficit disorder) or ADHD (attention-deficit hyperactivity disorder) symptoms. Currently, increased community concern about the prevalence, cost and impact of ADHD is evident in Australia, with a Senate inquiry recently announced. Such political recognition is likely to reflect in part the distinct prevalence of the condition, and with a recent Australian guideline document reporting an ADHD prevalence of 6%–10% in Australian children and adolescents and 2%–6% in adults.^1^ An economic analysis^2^ indicated that some 800,000 Australians were affected with ADHD and that the condition resulted in 12.83 billion dollars/year in direct costs, while multiple indirect costs were also detailed. That report detailed prevalence rates across age bands with the highest rates occurring for those in the 5-to 29-year bands and with prevalence rates dropping distinctly and linearly in older age bands. While such a pattern could reflect better awareness and diagnosis in younger individuals, it could also reflect a cohort effect whereby ADD/ADHD is now distinctly higher in children, adolescents and young adults.

Two (albeit US) studies quantifying increases in rates are noted. Chung et al.^3^ evaluated medical records of more than five million Californians and reported an increase from 2007 to 2016 in clinician-diagnosed incidence and prevalence rates of 43% and 123%, respectively. Xu et al.^4^ undertook a population-based survey of parents reporting that their child had received an ADD/ADHD diagnosis and quantified a linear increase in rates of the conditions being diagnosed over a 20-year period (from 6.1% in 1997–98 to 10.2% in 2015–16).

Such data and observations by clinical psychiatrists could reflect changed diagnostic criteria. DSM-III first listed ‘attention-deficit disorder’ and allowed two principal sub-types (‘with’ and ‘without’ hyperactivity) as well as a ‘residual type’ where hyperactivity once existed but was no longer present. The subsequent DSM-III-R manual listed a singular ADHD condition with criteria defining inattention, impulsiveness and hyperactivity. DSM-IV and DSM-5 allowed for (i) predominantly inattentive, (ii) predominantly hyperactive/impulsive and (iii) combined types and listed identical criteria sets. While DSM-IV had a criterion requiring there to be several symptoms present prior to 7 years of age, the relevant DSM-5 criterion was for symptoms to be present prior to 12 years of age. Such minor changes evidenced across the decades from DSM-III to DSM-5 argue against any increased prevalence reflecting a major change in diagnostic definition.

Other explanations of any temporal change could (a) reflect new ‘variants’ of the conditions emerging, (b) be a referral bias perhaps reflecting greater awareness of the conditions by those in the community or of individuals with the condition and so leading to an increase in clinical referral rates, (c) reflect milder versions previously remaining undetected till adulthood and/or (d) reflect there being a true increase in incidence.

‘Late-onset ADHD’ is generally conceptualized as capturing those with clear-cut ADHD in adulthood but without any salient criteria in childhood. Its status is informal as DSM-5 requires (as noted) symptoms to have been present ‘prior to age 12 years’. In a longitudinal New Zealand study, Moffit et al.^5^ reported that study participants having such a sub-type did not show neuropsychological deficits in childhood and suggested that aetiological factors in play for this variant differed from ones contributing to childhood onset ADHD. Sibley et al.^6^ undertook a survey of adults lacking ADHD criteria in childhood but meeting some criteria in adulthood. They judged that their sample members were more likely to have non-impairing cognitive fluctuations, a comorbid disorder or were experiencing cognitive effects of substance use, allowing that late-onset ADHD might be ‘pseudo-ADHD’.

I now offer some recent clinical observations. Only a minority of adults whom I suspect as having late-onset ADHD affirm classic features during primary school (e.g. being inattentive, highly distractible, disruptive and hyperactive) or over-represented features (e.g. inability to read a book and^7^ being the ‘class clown’^8^). The majority report being able to read without difficulty in primary school and, if then distractible or inattentive, judge such features as minor. In secondary school, they rarely returned low grades and only report their suspicion of ADHD arising from difficulty with educational assignments or occupational tasks. They rarely show distinct hyperactivity during interview but commonly report being generally fidgety, inattentive and distractible. Most are capable of sitting through a film at a theatre but tend to scan when reading, and if watching television, report attentional difficulty, generally having frequent breaks to wander around the room or access their phone multiple times.

Those in the late-onset group also generally report a distinct improvement in concentration and attention with a stimulant, which might confirm an ADD/ADHD diagnosis or simply reflect general benefits (improved concentration, task organization, focus and attention) experienced by those who do not have ADD or ADHD.

If late-onset ADD/ADHD is a valid condition what might be the aetiological candidates? ‘True’ ADD/ADHD has high hereditability, with a meta-analysis by Nikolas and Burt^9^ quantifying genetic factors as accounting for 71% and 73% respectively, in relation to inattention and hyperactivity, and with environmental factors contributing more to inattention than to hyperactivity. What environmental factors might be in play? Clinical observation suggests that most such patients are dogged mobile technology users.

So, is digital media involvement a precipitant of ‘true’ late onset ADHD? Or is reverse causality in play, whereby those with ADHD are more likely to so engage in such activities, or is there a bidirectional process as suggested by Gentile et al.^10^ In support of the primary hypothesis, Nikkelen et al.^11^ undertook a meta-analysis and quantified a moderate association between frequency of use of traditional forms of digital media (e.g. TV and video games) and ADHD symptoms. Clarification of the primary postulate requires a longitudinal study design, however, and one exemplar will be noted. Ra et al.^12^ recruited fifteen-year-old and sixteen-year-old adolescents lacking ‘significant symptoms of ADHD at baseline’ (p 255) and undertook multiple follow-up evaluations. At the 2-year reviews, those with low-frequency digital media use had a 4.6% rate of ADHD symptoms as against rates of 9.5% in those who engaged in high-frequency use and 10.5% in those in even higher-frequency use.

Experiments pursuing the proposition are increasing, albeit indirect ones. For example, Kushley et al.^13^ recruited general population volunteers to engage in 1 week of maximum and 1 week of minimum smart phone activity, with levels of both inattention and hyperactivity higher in the former phase.

Observationally, adolescents appear preoccupied with their mobile phones while formal reports support high usage. A US study quantified that 8–12 year olds and 13–18 year olds used screens for an average of 5.5 and 8.7 h each day, respectively.^14^ The Australian Government’s Australian Communications and Media Authority reported that, in 2020, nearly half (i.e. 46%) of Australian children aged 6–13 used a mobile phone.^15^ An Australian study^16^ quantified that child aged 12–13 averaged 2.2 h of screen time on school days and 4.2 h on weekends.

Thus, rather than distinguish between ‘standard’ and late-onset ADD/ADHD, it may be preferable to conceive of ‘inherent’ and ‘acquired’ conditions. Those in the first group would be more likely to report a family history, evidence symptoms in primary school and generally have a more severe expression of the condition than those with a late onset and who would rarely report a family history. Confirmation of such a model would allow important research. It might be hypothesized that those with inherent ADD/ADHD would respond preferentially to a stimulant, while those with acquired ADD/ADHD – who might also benefit from such medication – would also report some improvement with lifestyle changes that reduce their digital involvement. Standard measures of ADD/ADHD status may need to be modified to capture both expressions across childhood, adolescence and early adulthood. Alternatively, an epigenetic mechanism may be in play with such environmental changes increasing ADD/ADHD symptoms, especially when it is recognized that environmental changes can improve ADHD symptoms.^17^

In a recently published book, sub-titled ‘Why we need to transform how the world sees time’, Richard Fisher^18^ noted that we have become a society trapped by a ‘tyranny of the instant and the treadmill of the unending now’. Digital pre-occupation and increasing pressures for a 24/7 online presence may be setting a variant trap and springing a new variant of ADD/ADHD into existence.
